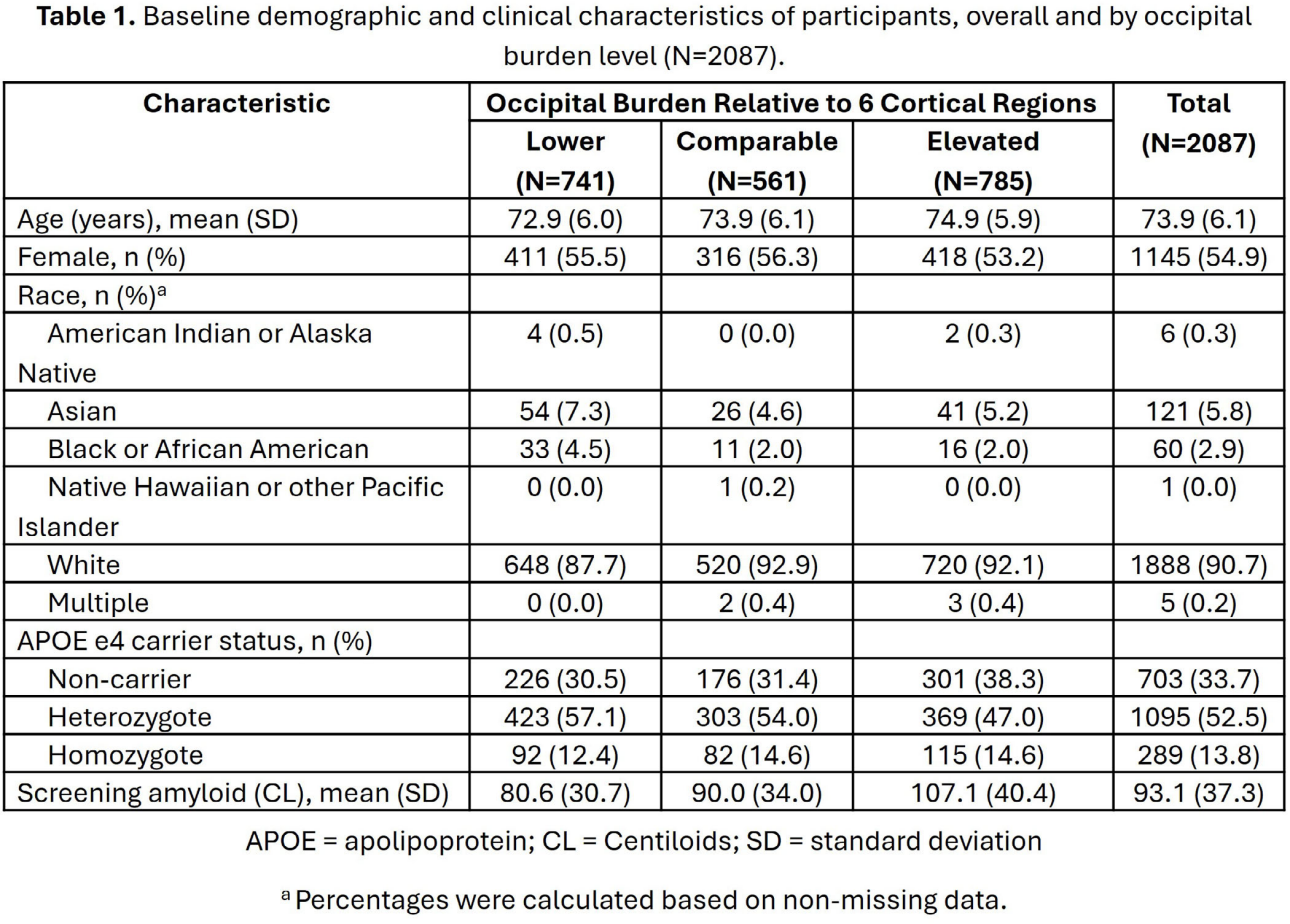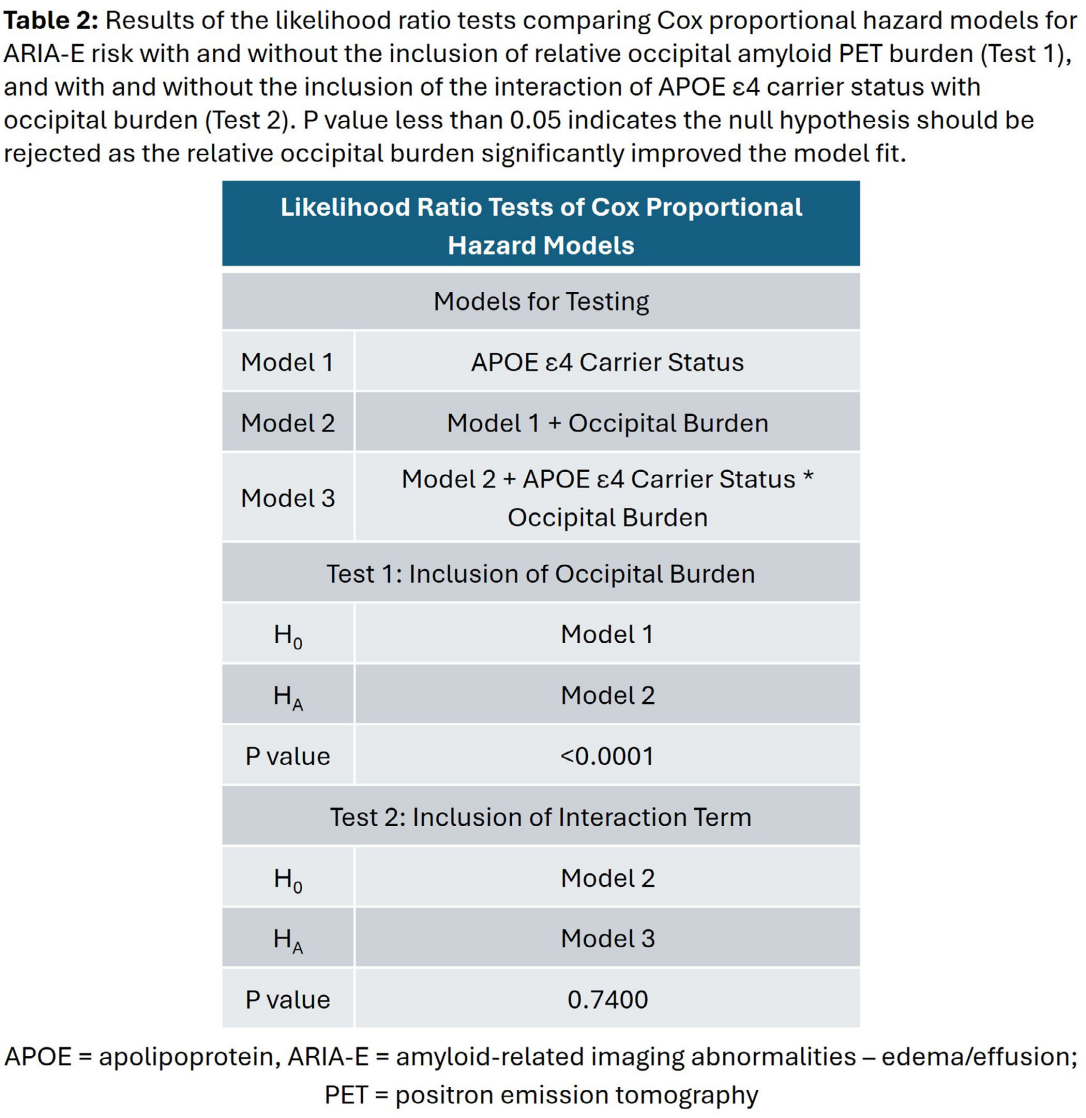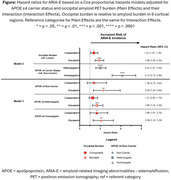# Association of Occipital Amyloid PET Burden with ARIA‐E: Exploratory Analyses in Three Clinical Trials of Donanemab

**DOI:** 10.1002/alz70862_110828

**Published:** 2025-12-23

**Authors:** Ian A. Kennedy, Min Jung Kim, Ming Lu, John R. Sims, Mark A. Mintun, Emily C. Collins, Sergey Shcherbinin

**Affiliations:** ^1^ Eli Lilly and Company, Indianapolis, IN USA

## Abstract

**Background:**

Amyloid‐related imaging abnormalities including edema/effusions (ARIA‐E) occur with amyloid‐targeting therapies. Major risk factors for ARIA‐E include apolipoprotein (APOE) ε4 carrier status, microhemorrhages, and cerebral amyloid angiopathy (CAA). Though differential diagnosis between CAA and Alzheimer’s disease (AD) is challenging, amyloid plaque burden in occipital regions may be higher in CAA than AD. This work explores the association of ARIA‐E with occipital amyloid burden in donanemab‐treated participants with early symptomatic AD.

**Method:**

Data included donanemab‐treated participants with early symptomatic AD in TRAILBLAZER‐ALZ (NCT03367403), TRAILBLAZER‐ALZ 2 (NCT04437511), and TRAILBLAZER‐ALZ 4 (NCT05108922). Amyloid pathology was assessed with florbetapir or florbetaben positron emission tomography (PET). Baseline amyloid burden was calculated in the atlas‐based lateral occipital region referenced to the whole cerebellum. Participants were categorized as having lower, comparable, or elevated occipital uptake relative to 6 cortical regions included in global amyloid assessments; these thresholds were derived from the Imaging Dementia‐Evidence for Amyloid Scanning Study (NCT02420756) after region‐ and tracer‐specific pre‐processing. The association of baseline occipital burden level with ARIA‐E over 76 weeks was assessed with Cox proportional hazard modeling adjusted for APOE ε4 carrier status and their interaction.

**Result:**

Analysis included *N* = 2087 baseline amyloid PET scans, with 741, 561, and 785 scans with lower, comparable, and elevated occipital amyloid burden relative to 6 cortical regions, respectively (Table 1). Baseline occipital burden was independently associated with ARIA‐E over 76 weeks (Table 2, Model 2). Compared with lower occipital burden, both comparable and elevated occipital burden were associated with statistically significantly higher risk of ARIA‐E, even when accounting for APOE ε4 carrier status (Figure, Model 2). Though the interaction of occipital burden with APOE ε4 status was not statistically significant (Table 2, Model 3), elevated occipital burden was associated with ARIA‐E within all APOE ε4 carrier groups (Figure, Model 3).

**Conclusion:**

Elevated occipital amyloid burden at baseline was statistically significantly associated with risk of ARIA‐E in donanemab‐treated participants with early symptomatic AD. The potential of occipital burden to predict ARIA‐E should be further validated with additional methodologies, subgroup analyses, and datasets.